# Convergent ethical issues in HIV/AIDS, tuberculosis and malaria vaccine trials in Africa: Report from the WHO/UNAIDS African AIDS Vaccine Programme's Ethics, Law and Human Rights Collaborating Centre consultation, 10-11 February 2009, Durban, South Africa

**DOI:** 10.1186/1472-6939-11-3

**Published:** 2010-03-09

**Authors:** Nicole Mamotte, Douglas Wassenaar, Jennifer Koen, Zaynab Essack

**Affiliations:** 1 African AIDS Vaccine Programme's Ethics, Law and Human Rights Collaborating Center, School of Psychology, University of KwaZulu-Natal, Pietermaritzburg, South Africa

## Abstract

**Background:**

Africa continues to bear a disproportionate share of the global HIV/AIDS, tuberculosis (TB) and malaria burden. The development and distribution of safe, effective and affordable vaccines is critical to reduce these epidemics. However, conducting HIV/AIDS, TB, and/or malaria vaccine trials simultaneously in developing countries, or in populations affected by all three diseases, is likely to result in numerous ethical challenges.

**Methods:**

In order to explore convergent ethical issues in HIV/AIDS, TB and malaria vaccine trials in Africa, the Ethics, Law and Human Rights Collaborating Centre of the WHO/UNAIDS African AIDS Vaccine Programme hosted a consultation on the *Convergent Ethical Issues in HIV/AIDS, TB and Malaria Vaccine Trials in Africa *in Durban, South Africa on the 10-11 February 2009.

**Results:**

Key cross cutting ethical issues were prioritized during the consultation as community engagement; ancillary care obligations; care and treatment; informed consent; and resource sharing.

**Conclusion:**

The consultation revealed that while there have been few attempts to find convergence on ethical issues between HIV/AIDS, TB and malaria vaccine trial fields to date, there is much common ground and scope for convergence work between stakeholders in the three fields.

## Background

Africa continues to bear a disproportionate share of the global burden of HIV, TB and malaria. There are 22 million people living with HIV in sub-Saharan Africa, 67% of the 33 million people living with HIV globally [1]. An estimated 9.27 million new cases of TB [2] and 1.7 million deaths from TB [3] occurred globally in 2007. Thirteen of the 15 countries with the highest estimated TB incidence rates are in Africa, a result of high rates of HIV co-infection [2]. In 2006, there were an estimated 247 million episodes of malaria and an estimated 881 000 malaria deaths [4]. Eighty-six percent (212 million) of these malaria episodes and 91% (801 000) of malaria deaths in 2006 occurred in Africa (Ibid.). Malaria and TB complicate the effective control of HIV, due to their shared risk factors, geographic overlap and co-infection, particularly in sub-Saharan Africa. TB is a leading cause of death among people living with HIV/AIDS in Africa [5]. Only 1% of people living with HIV/AIDS are reported to have been screened for TB, of which more than 25% have TB [6]. HIV also increases the risk of malaria infection and the development of clinical malaria, while malaria has been shown to induce HIV-1 replication and increase HIV viral load [7].

Despite the variety of prevention, treatment and care initiatives in existence, significant challenges in curbing these diseases remain. The development and distribution of vaccines that are safe, effective and affordable is critical to reduce disease and mortality from HIV/AIDS, TB, and malaria. The geographic overlap, shared risk factors and possibility for co-infection between HIV/AIDS, TB, and malaria mean that these diseases can no longer be considered in isolation. International cooperation and the development and testing of multiple vaccines simultaneously, are required to curb these epidemics.

The last few years have seen significant progress in the development of vaccines against HIV/AIDS, TB, and malaria. Despite several setbacks, the search for a safe and effective HIV/AIDS vaccine continues. There are presently HIV/AIDS vaccine trials being conducted in four African countries namely Uganda, Kenya, Tanzania and South Africa [8]. Proof-of-concept of vaccine-induced protection from malaria infection and disease has recently been demonstrated in African children and several candidate malaria vaccines are in early clinical trials [9]. In Africa, malaria vaccine clinical trials are ongoing in Mozambique, Tanzania, Gabon, Ghana, Kenya, the Gambia, Burkina Faso and Mali (Ibid.). While a TB vaccine, Bacillus Calmette-Guérin (BCG), is available, it provides approximately 80% protection against TB meningitis and miliary TB in infancy and in young children but provides only variable protection against lung disease, at all ages, creating an urgent need for new candidate vaccines [10]. In Africa, TB vaccine trials are being conducted in South Africa and the Gambia (Ibid.), with site development taking place in Kenya, Uganda, Mozambique and Tanzania.

Conducting vaccine trials in developing countries is ethically complex and requires careful consideration of several key issues [11]. HIV/AIDS, TB, and malaria vaccine trials each present unique ethical challenges. HIV/AIDS vaccine trial participants, for example, may experience discrimination accorded to individuals perceived to be at high risk of HIV/AIDS or as a result of false seropositivity on conventional HIV testing methods [12]. In Malaria vaccine research the ethics of testing transmission-blocking vaccines that do not protect vaccinees from infection or disease but prevent infection of others, needs careful consideration [13]. In TB vaccine research, the use of placebo controlled trials may be considered unethical as standard BCG vaccines (although not reliably efficacious for TB prevention) are widely used and as such could, depending on the characteristics of the study population, be provided to control groups [14]. In addition to such challenges, conducting HIV/AIDS, TB, and/or malaria vaccine trials simultaneously in developing countries, or in populations affected by all three diseases, is likely to result in numerous other ethical challenges.

## Methods

In order to explore convergent ethical issues in HIV/AIDS, TB and malaria vaccine trials in Africa, the Ethics, Law and Human Rights Collaborating Centre of the WHO/UNAIDS African AIDS Vaccine Programme hosted a consultation on the *Convergent Ethical Issues in HIV/AIDS, TB and Malaria Vaccine Trials in Africa *in Durban, South Africa on the 10-11 February 2009. This meeting aimed to bring together key stakeholders from HIV/AIDS, TB, and malaria vaccine initiatives to explore the convergent ethical issues in the three fields and to promote and impact cooperation, networking and resource sharing in Africa. Participants included sponsors, investigators, research ethics committee members and community representatives working in the fields of HIV/AIDS, TB and malaria vaccine research. This paper outlines the key convergent ethical issues and recommendations identified during the consultation.

## Results and discussion

### Ethical issues in HIV, TB and Malaria vaccine trials

Using an informal ranking exercise, the key cross cutting ethical issues in HIV/AIDS, TB, and malaria vaccine research in Africa, were roughly prioritized by stakeholders in the consultation as follows,: 1) Community engagement; 2) Ancillary care obligations; 3) Care and treatment; 4) Informed consent; 5) Resource sharing. These are detailed below, with recommendations for each.

#### 1. Community Engagement

##### Identified Issues

Community engagement approaches appear to be better developed in HIV/AIDS research than in research relating to TB and malaria. HIV/AIDS research typically includes broad community education campaigns (which are not specific to the research study, but are about HIV/AIDS more generally) and community consultation regarding specific studies. Conversely, TB related trials have, in general, not included such community involvement and preparation for research. This might be argued to be a result of the global advocacy around HIV/AIDS (and the fact that unlike malaria and TB, HIV/AIDS has had a marked impact on the developed world). HIV/AIDS advocacy in North America changed human rights globally. HIV/AIDS became more than a health-related issue and is now also a political issue. Despite this difference, stakeholders agreed that relationships with communities are an important ethical concern for all three fields and that community engagement needs to move beyond the tokenistic involvement of the community and towards more power sharing relationships. Community engagement is a critical component in clarifying reasonable expectations regarding study results (outcomes and access to products post-trial) and care, for managing issues around the therapeutic misconception and for ensuring that procedures are culturally sensitive. Community engagement is also increasingly desired and required when considering at what stage in trial planning to involve communities and how best to do so.

##### Recommendations

It is recommended that a science of community engagement be encouraged [15], where stakeholders place as much emphasis on researching and engaging with the community as they do on conducting trials and that negotiation skills be imparted to communities so that they can participate as active partners in the trial planning and process. It is also recommended that the UNAIDS Ethics Guidance [16] and Good Participatory Practice (GPP) [17] documents, developed for preventive HIV/AIDS research, be adapted and used by investigators in malaria and TB prevention initiatives.

#### 2. Ancillary Care Obligations

##### Identified Issues

Careful advanced planning on which ancillary diseases will be treated is essential [18-20]. Ancillary care is defined as care provided to clinical trial participants which goes beyond the requirements of scientific validity, safety, keeping promises, or rectifying injuries [18]. Investigators may perform ancillary care procedures if they are minor and will not impact on the study protocol. The provision of ancillary care is potentially problematic. For example, in cases where the ancillary care results in harm, what liability is there on the investigators or sponsors and what implications could the provision of ancillary care, even if protocol specific, have for study and investigator insurance? The provision of ancillary care may complicate the relationship between investigators and study participants. Participants may come to view the research relationship as therapeutic and may come to trust investigators to a degree where they unquestioningly accept investigative procedures [20]. The provision of ancillary care and the potential blurring of the investigative relationship with a therapeutic one may potentially exacerbate the therapeutic misconception [21]. This could be a particular concern for HIV/AIDS vaccine trials where the therapeutic misconception could potentially lead to behavioural disinhibition in some participants [22]. Furthermore, the provision of ancillary care in one study may lead to raised expectations of care in future studies.

##### Recommendations

It is recommended that the provision of ancillary care be carefully considered during protocol development, in consultation with host communities, and that responsibility for investigative and ancillary care procedures is effective, clearly differentiated and performed by designated or different members of the trial staff. It is also recommended that *a priori *partnerships be forged and formalized between local health service providers and researchers to develop a referral system for ancillary care prior to trial initiation so that participants can be referred for ancillary care, when appropriate.

#### 3. Care and Treatment

##### Identified Issues

Defining the standard of care and treatment to be provided when a participant becomes infected with the pathogen under study is a contentious issue. While TB and malaria are curable and treatments are relatively inexpensive and readily available, HIV/AIDS remains incurable and life-long treatment is required. Standards of care and treatment, particularly for HIV/AIDS, are usually much higher in sponsor countries (Europe and North America) and sponsors may require this higher standard in developing country settings when it is not the norm for the local population. This issue is a particular concern for multi-centre studies where the standard of care and treatment may vary across centres, or across participants and non-participants within a host country. Regardless of which standard is implemented, research sites generally provide better care than that which is provided by the health systems in the host country. Sustainability of this 'higher' standard of care and treatment beyond the duration of the study is a challenge faced by many investigators.

##### Recommendations

Clear boundaries regarding care and treatment obligations need to be delineated. In contexts where referral systems are well-developed and investigators can refer participants, follow-up is crucial as the ethical responsibility to participants does not end once referrals are made. Complex decisions on how care and treatment obligations should be met should be trial specific and made through an open and participatory process before a trial begins [16,23]. The type, range and duration of care and treatment provided, as well as who will finance and implement it, should also be decided on in advance (Ibid.).

#### 4. Informed Consent

##### Identified Issues

Factors such as poor health delivery systems, poverty, the therapeutic misconception and illiteracy may hinder the process of obtaining truly informed consent. In malaria and TB vaccine research, where target populations are often children, extra care and effort are needed on the part of research teams when obtaining proxy consent from parents/guardians, especially in cases where the parents may themselves be minors. Sponsors often require long, detailed and overly legalistic consent forms to be administered. These forms are intimidating and challenging to administer due to low levels of literacy among the majority of volunteers. As such, understanding may be limited. Therefore, local investigators argue for short, simplified versions of consent forms. Changing consent form templates involves significant power dynamics between sites and sponsors, and some sponsors may be unwilling to relinquish this. The debate about length, detail and usefulness of consent forms may be seen as a function of over-regulation of the clinical trials industry. Studies are often not approved if they do not include lengthy consent forms, regardless of their appropriateness. This may be reflective of assumptions that mere compliance with regulations regarding what to include in consent forms is equal to meeting certain ethical standards, which is not necessarily the case.

##### Recommendations

Sharing of simple and effective consent form templates and procedures across disease areas is recommended [24] as is the need to focus on the 'process' of consent and supplementary consent materials, rather than only on the actual form, in order to make meaningful consent more possible. It is also recommended that comprehension tests be administered to participants prior to and during participation in clinical trials to ensure that they understand important trial concepts. Community Advisory Groups should also be educated on clinical trial procedures so that they can review the contents of the consent forms and materials [17].

#### 5. Resource Sharing

##### Identified Issues

Collaborative relationships between researchers and research centres, working within, and across, each of the disease entities is critical in promoting infrastructural and capacity development in developing countries, and in responding to ethical challenges. Working in pathogen-specific silos is an artifact, as TB, HIV/AIDS and malaria are related and are often co-morbid, and communities may want attention paid to the spectrum of diseases affecting them. Attempts to diversify across fields should acknowledge that working in silos does tend to serve donor priorities and thus researchers' personal career interests, particularly in light of competition for funding. Sharing of resources and capacity across disease areas may however help to avoid wasting capacity, (e.g., clinical trial capacity, laboratory capacity) which has been established in research teams, between trials, and when candidate products are unavailable. Potential barriers to resource sharing include institutional separation, ignorance of each field's history and unique challenges, constrained resources and different timelines. Potential enablers include identifying common platforms of issues and developing models and tools that can serve more than one initiative. The principal advantages are the maximisation of finite resources and energies, and the promotion and retention of local capacity.

##### Recommendations

Given the limited resources and capacity in Africa, it is recommended that where capacity has been developed, and where sophisticated infrastructure has been put in place, within a disease entity, this should be shared within and across disease entities in order to further the objective of addressing health and development at a broader level. This would include the sharing of training resources (around ethics, research conduct and community research literacy) across disease entities. This would also enable the retention of scarce skills and reduce 'brain drain' from the developing world.

## Conclusion

The consultation revealed that while there have been few attempts to find convergence on ethical issues between HIV/AIDS, TB and malaria vaccine trial fields to date, there is much common ground and scope for convergence work between stakeholders in the three fields. Based on recommendations made during the consultation, a steering committee has been established and possible funding sources have been identified to move the convergence agenda forward. Additional activities recommended to move the convergence agenda forward include: An empirical study of how key stakeholders in HIV/AIDS, TB and malaria vaccine trials would prioritise convergent ethical issues; the documentation of the existence of disease specific 'silos'; a study of knowledge differences and barriers to convergence between the three fields; and the hosting of a larger forum, with better representation from the key stakeholders and decision makers in the three fields, to debate these issues further.

## Abbreviations

AIDS: Acquired Immune Deficiency Syndrome; HIV: Human Immunodeficiency Virus; TB: Tuberculosis; UNAIDS: Joint United Nations Programme on HIV/AIDS.

## Competing interests

The authors declare that they have no competing interests.

## Authors' contributions

DW chaired the consultation and NM, JK and ZE rapporteured the consultation to which the manuscript refers. All authors drafted the manuscript and read and approved the final manuscript.

## Authors' information

NM is the Project Manager of the African AIDS Vaccine Programme's Ethics, Law and Human Rights Center, School of Psychology, University of KwaZulu-Natal.

DW is a Professor at the School of Psychology, University of KwaZulu-Natal, South Africa and the Principal investigator of the South African Research Ethics Training Initiative and the WHO-UNAIDS African AIDS Vaccine Programmes' Ethics, Law and Human Rights Centre, School of Psychology, University of KwaZulu-Natal.

JK and ZE are researchers at the South African AIDS Vaccine Initiative's HIV AIDS Vaccine Ethics Group, School of Psychology, University of KwaZulu-Natal.

## Pre-publication history

The pre-publication history for this paper can be accessed here:

http://www.biomedcentral.com/1472-6939/11/3/prepub
